# Supplementary material for: Effect of high-frequency low-intensity pulsed electric field on protecting SH-SY5Y cells against hydrogen peroxide and β-amyloid-induced cell injury via ERK pathway
Source: PLoS One. 2021 Apr 26;16(4):e0250491. doi: 10.1371/journal.pone.0250491 (PMC8075192; doi:10.1371/journal.pone.0250491)
Supplement: S1 File — (PDF) [file pone.0250491.s001.pdf]

Fig 2A

H2O2 (uM)	0.000	100.000	200.000	300.000	400.000	500.000
	101.686	98.227	86.051	75.563	64.833	54.878
	98.863	98.752	86.051	74.397	68.625	53.177
Normalized	99.450	88.200	88.507	79.188	64.446	53.390
Viability	100.228	88.929	83.189	78.452	65.162	53.390
average	100.000	95.060	86.870	76.383	65.968	53.815

Fig 2B

PEF(Hz)	CTRL	H2O2	2.000	10.000	50.000	100.000
	99.499	53.390	49.835	55.369	50.389	59.451
Normalized	99.181	53.177	51.161	50.855	63.948	61.736
Viability	101.321	52.174	54.300	58.378	63.799	60.380
average	100.000	52.914	51.765	54.867	59.379	60.522
PEF(Hz)	200.000	400.000	499.000			
	72.115	56.356	57.394			
Normalized	79.039	63.182	50.401			
Viability	70.876	66.949	56.979			
average	74.010	62.162	54.924			

Fig 2C

PEF(V/cm)	CTRL	H2O2	1.000	5.000	10.000	20.000
	103.630	54.878	53.036	58.376	67.017	62.462
Normalized	96.120	48.101	51.996	66.372	70.876	56.638
Viability	100.251	54.740	57.857	59.005	79.039	69.887
average	100.000	52.573	54.296	61.251	72.311	62.996
PEF(V/cm)	25.000	30.000	45.000	60.000		
	55.187	61.007	52.340	58.352		
Normalized	52.181	50.407	57.077	56.351		
Viability	62.207	59.788	50.491	48.349		
average	56.525	57.068	53.303	54.351		

Fig 2D

	CTRL	H-LIPEF	H2O2	H-LIPEF+H2O2
	97.229	105.131	46.101	74.215
Normalized	101.086	103.630	55.740	71.247
Viability	101.686	96.120	54.545	79.039
average	100.000	101.627	52.129	74.834

Fig 3B

	CTRL	H-LIPEF	H2O2	H-LIPEF+H2O2
	4.400	8.300	38.700	26.900
Apoptotic	6.200	9.000	34.200	17.800
cells (%)	3.200	5.900	33.200	17.500
average	4.600	7.733	35.367	20.733

Fig 4B

	CTRL	H-LIPEF	H2O2	H-LIPEF+H2O2
	102.985	102.705	50.653	76.959
Normalized	100.746	102.705	54.291	73.041
Viability	96.269	100.466	50.373	85.354
average	100.000	101.959	51.772	78.451

Fig 5A

Abeta (uM)	CTRL	25.000	50.000
	106.590	69.037	56.307
Normalized	94.269	70.605	60.862
Viability	99.140	65.458	63.441
average	100.000	68.367	60.203

Fig 5B ThT

	ThT	ThT+Abeta
ThT	0.949	10.096
fluorescence	1.000	8.874
intensity	1.051	7.389
average	1.000	8.786

Fig 5C

	CTRL	H-LIPEF	Abeta	H-LIPEF+Abeta
	91.339	103.543	64.567	83.465
Normalized	103.543	88.976	60.142	89.370
Viability	105.118	105.118	59.929	77.953
average	100.000	99.213	61.546	83.596

Fig 5E

	CTRL	H-LIPEF	H2O2	H-LIPEF+H2O2
	62.730	56.922	22.846	58.470

cell numbers	55.566	68.345	16.844	46.467
($x10^3/mm^2$)	56.728	60.407	19.555	51.888
average	58.341	61.891	19.748	52.275

Fig 6B

	CTRL	H-LIPEF	H2O2	H-LIPEF+H2O2
ROS level	100.000	105.836	181.167	114.324
(% of	100.000	101.563	159.375	121.484
control)	100.000	103.191	191.578	113.209
average	100.000	103.530	177.373	116.339

Fig 7B

	CTRL	H-LIPEF	H2O2	H-LIPEF+H2O2
Cells with	36.400	34.500	62.600	38.200
decreased	38.000	36.100	53.400	42.500
MMP (% of	35.700	40.200	78.600	34.700
average	36.700	36.933	64.867	38.467

Fig 8B

			ROCK/GAPDH	
	CTRL	PEF	H2O2	H-LIPEF+H2O2
	100.000	99.171	115.347	116.357
	100.000	91.634	93.720	96.269
Normalized	100.000	91.866	106.933	109.071
Viability	100.000	95.503	112.041	84.859
average	100.000	94.544	107.010	101.639

Fig 9

			p-ERK/GAPDH	
	CTRL	PEF	H2O2	H-LIPEF+H2O2
	100.000	85.104	39.130	132.287
p-ERK/GAP	100.000	80.141	34.325	94.818
	100.000	92.757	36.486	95.545
average	100.000	86.001	36.647	107.550

			t-ERK/GAPDH	
	CTRL	PEF	H2O2	H-LIPEF+H2O2
t-ERK/GAP	100.000	88.330	53.721	94.086
	100.000	99.082	69.418	99.135
	100.000	72.976	67.336	114.863
average	100.000	86.796	63.492	102.695

			p-ERK/t-ERK	
	CTRL	PEF	H2O2	H-LIPEF+H2O2
p-ERK/t-ERK	100.000	117.746	69.598	133.147
	100.000	127.106	54.185	85.468
	100.000	110.795	52.621	89.212
average	100.000	118.549	58.801	102.609

			Nrf2/GAPDH	
	CTRL	PEF	H2O2	H-LIPEF+H2O2
Nrf2/GAPDH	100.000	111.336	71.114	109.659
	100.000	130.067	69.618	112.902
	100.000	100.340	39.174	84.001
	100.000	91.996	80.973	111.532
average	100.000	113.914	59.969	102.187

			p-CREB/GAPDH	
	CTRL	PEF	H2O2	H-LIPEF+H2O2
p-CREB/GAP	100.000	84.984	8.493	48.297
	100.000	87.946	8.803	58.759
	100.000	67.230	12.341	50.491
average	100.000	80.053	9.879	52.516

			t-CREB/GAPDH	
	CTRL	PEF	H2O2	H-LIPEF+H2O2
t-CREB/GAP	100.000	97.828	32.758	73.407
	100.000	103.300	39.383	107.909
	100.000	79.206	25.858	63.922
average	100.000	93.444	32.666	81.746

	CTRL	PEF	p-CREB/t-CREB<br>H2O2	H-LIPEF+H2O2
	100.000	86.871	25.927	65.794
p-CREB/t-CREB	100.000	85.136	22.353	54.452
	100.000	84.881	47.727	78.989
average	100.000	85.629	32.002	66.412

	CTRL	PEF	BCL-2/BAX<br>H2O2	H-LIPEF+H2O2
	100.000	120.216	71.754	103.818
	100.000	125.228	73.447	115.114
BCL-2/BAX	100.000	128.109	75.700	91.600
average	100.000	124.518	73.634	103.511

Fig 10A

	CTRL	H2O2	PD	H-LIPEF+H2O2	PD+LIPEF+H2O2
	99.231	58.935	83.579	79.527	54.497
Normalized	100.296	55.385	100.566	85.207	59.467
Viability	100.473	58.225	93.092	68.876	55.740
average	100.000	57.515	92.412	77.870	56.568

Fig 10B

	CTRL	H2O2	p-ERK/GAPDH<br>PD	H-LIPEF+H2O2	PD+LIPEF+H2O2
	100.000	52.149	31.964	87.224	32.475
p-ERK/GAP	100.000	42.086	31.343	99.029	36.309
	100.000	51.643	25.209	96.827	37.380
average	100.000	48.626	29.505	94.360	35.388

	CTRL	H2O2	t-ERK/GAPDH<br>PD	H-LIPEF+H2O2	PD+LIPEF+H2O2
	100.000	79.377	177.093	86.700	221.245
t-ERK/GAP	100.000	72.766	209.034	88.371	236.696
	100.000	69.483	121.202	106.672	201.926
average	100.000	73.875	169.110	93.914	219.956

			p-ERK/t-ERK		
	CTRL	H2O2	PD	H-LIPEF+H2O2	PD+LIPEF+H2O2
p-ERK/t-ERK	100.000	64.749	16.036	104.155	16.259
	100.000	63.931	14.741	134.655	17.796
	100.000	51.297	20.799	90.771	18.512
average	100.000	59.992	17.192	109.860	17.522
