# Supplementary material for: Effect of high-frequency low-intensity pulsed electric field on protecting SH-SY5Y cells against hydrogen peroxide and β-amyloid-induced cell injury via ERK pathway
Source: PLoS One. 2021 Apr 26;16(4):e0250491. doi: 10.1371/journal.pone.0250491 (PMC8075192; doi:10.1371/journal.pone.0250491)
Supplement: S2 File — (PDF) [file pone.0250491.s002.pdf]

Fig 8A

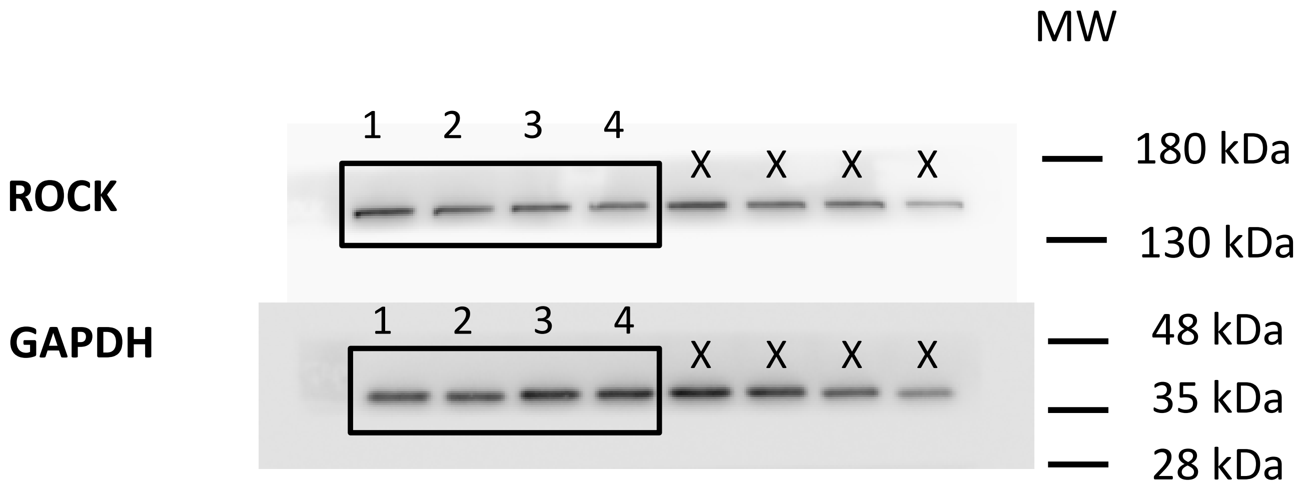

1: CTRL
2: H-LIPEF
3: H_2O_2
4: H-LIPEF+H_2O_2

The uncropped images of the western blot results are provided in this document. The strips of the protein bands were visualized with an enhanced chemiluminescence substrate (Advansta, Inc.) and detected with the Amersham Imager 600 imaging system (GE Healthcare Life Sciences).

Fig 9A

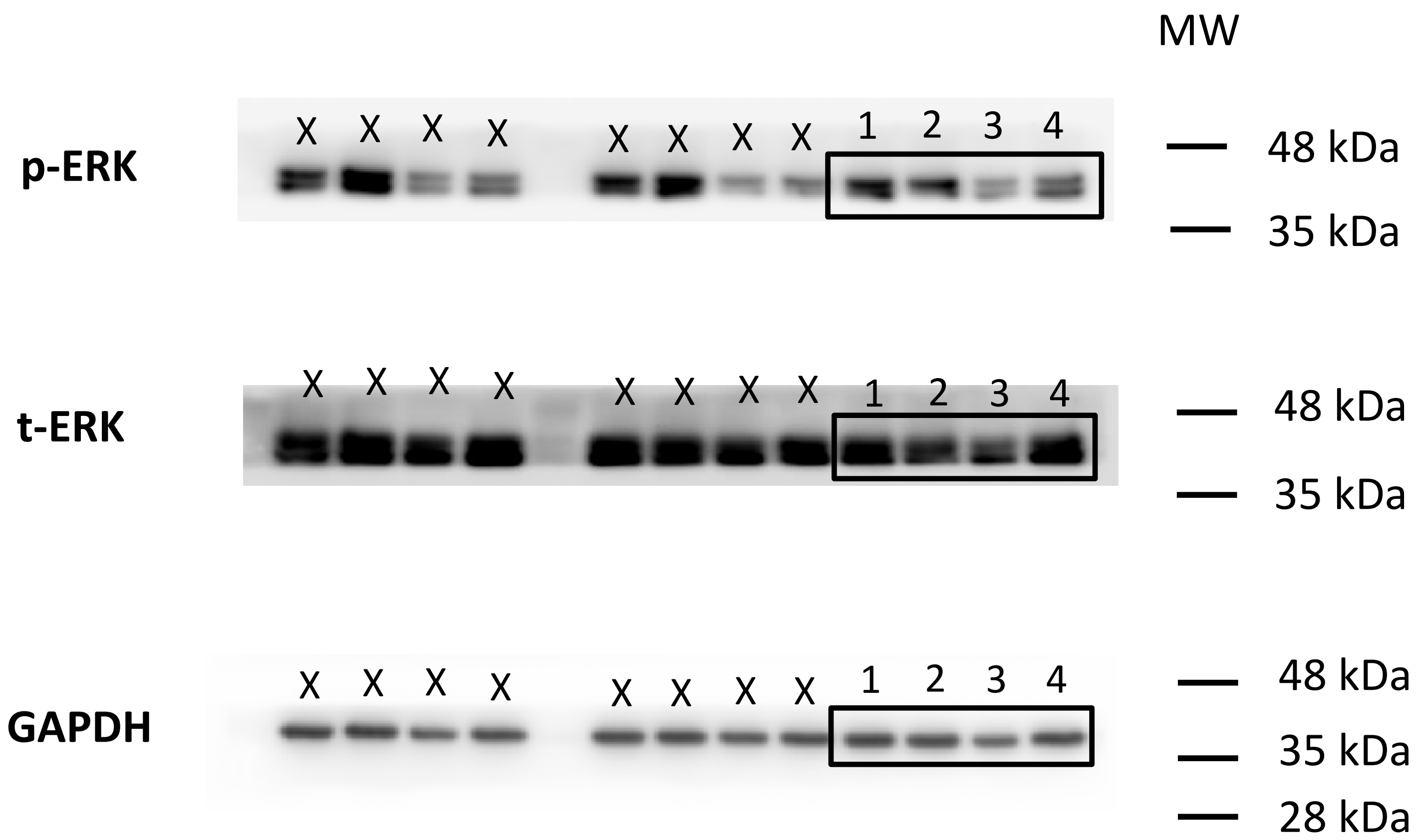

1: CTRL
2: H-LIPEF
3: H_2O_2
4: H-LIPEF+H_2O_2

Fig 9B

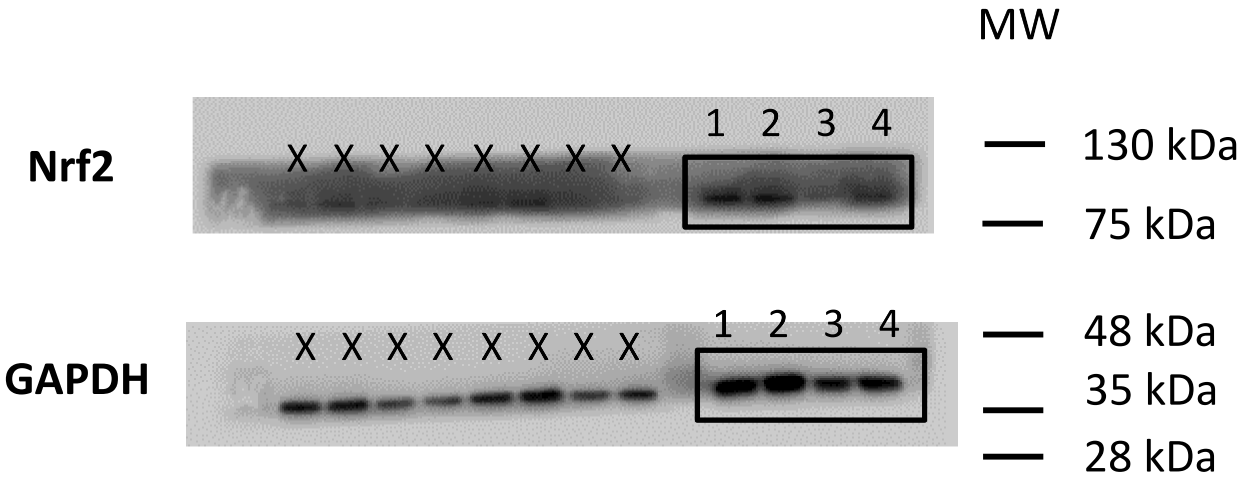

1: CTRL
2: H-LIPEF
3: H_2O_2
4: H-LIPEF+H_2O_2

Fig 9C

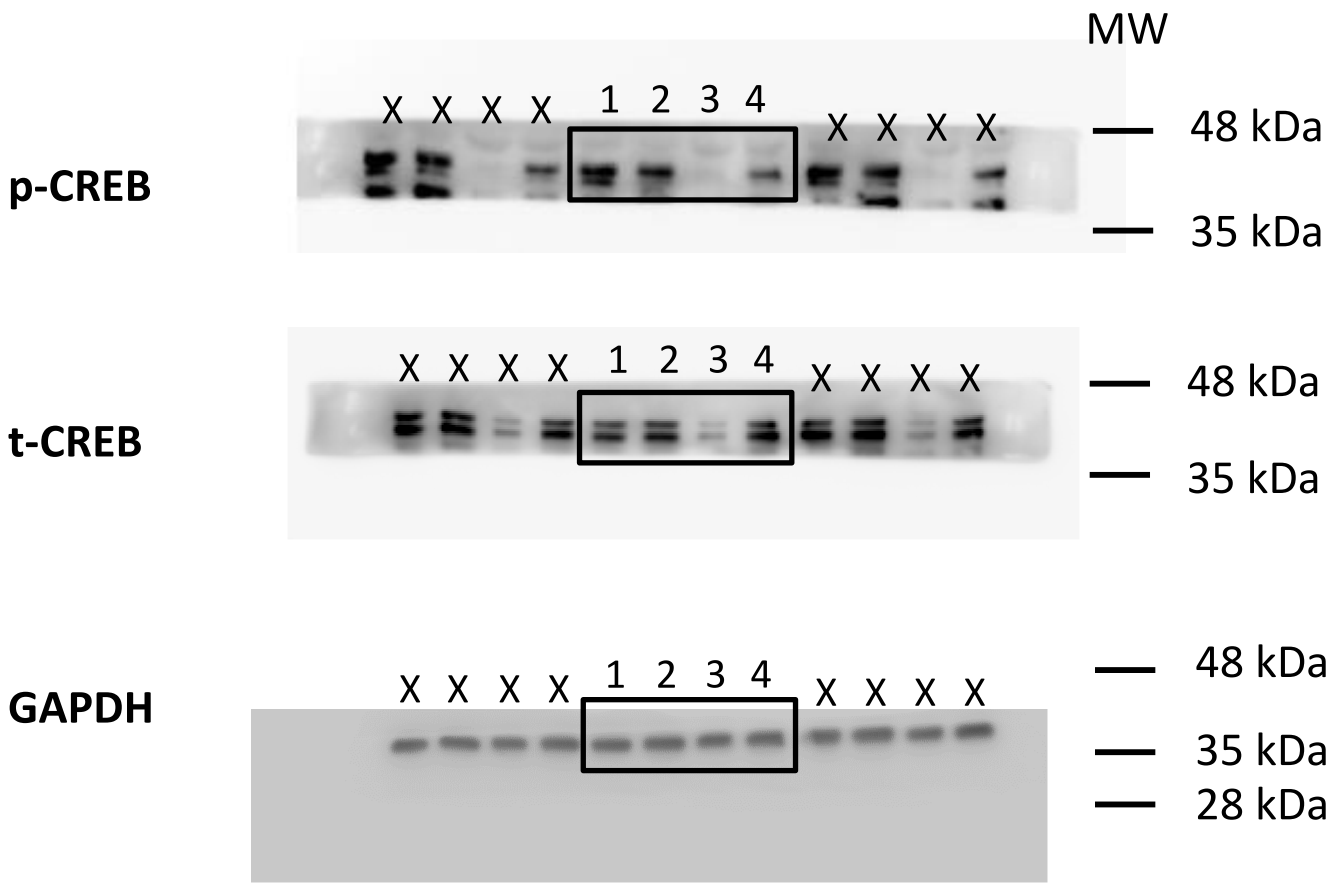

1: CTRL
2: H-LIPEF
3: H_2O_2
4: H-LIPEF+H_2O_2

Fig 9D

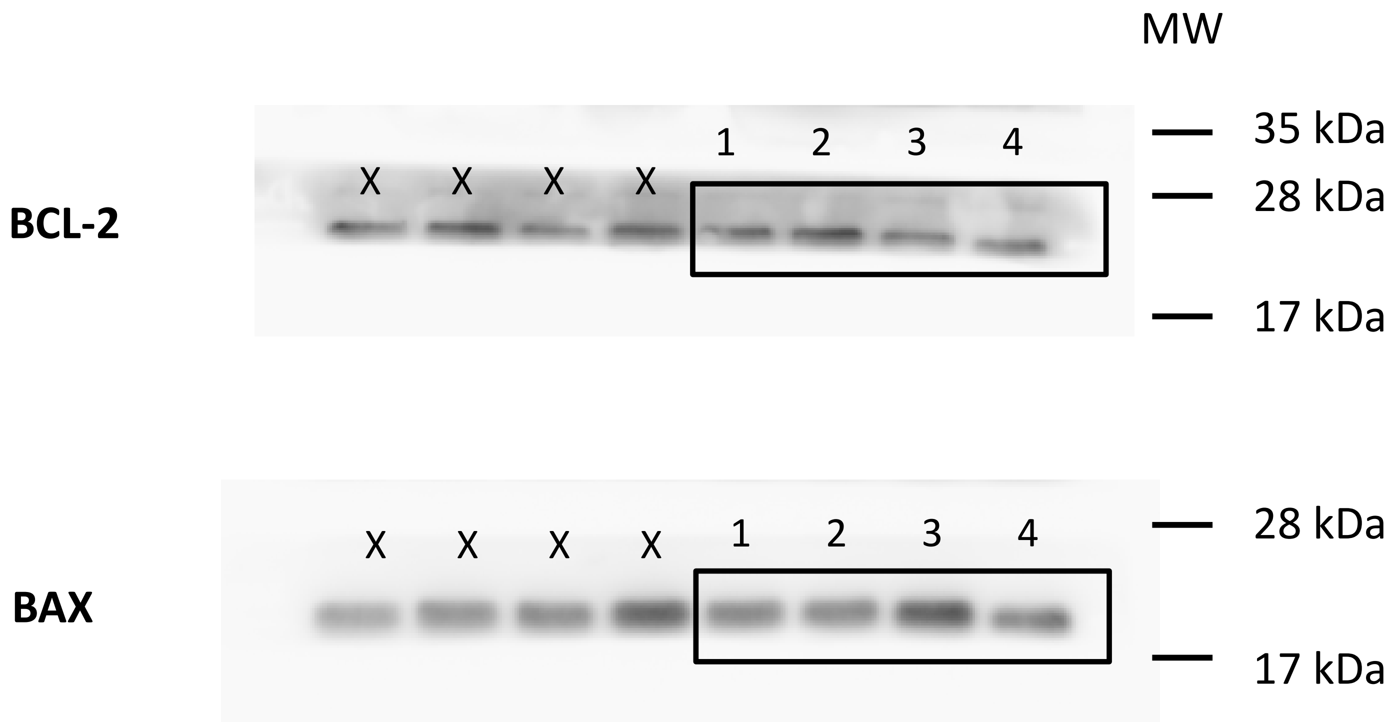

1: CTRL
2: H-LIPEF
3: H_2O_2
4: H-LIPEF+H_2O_2

Fig 10B

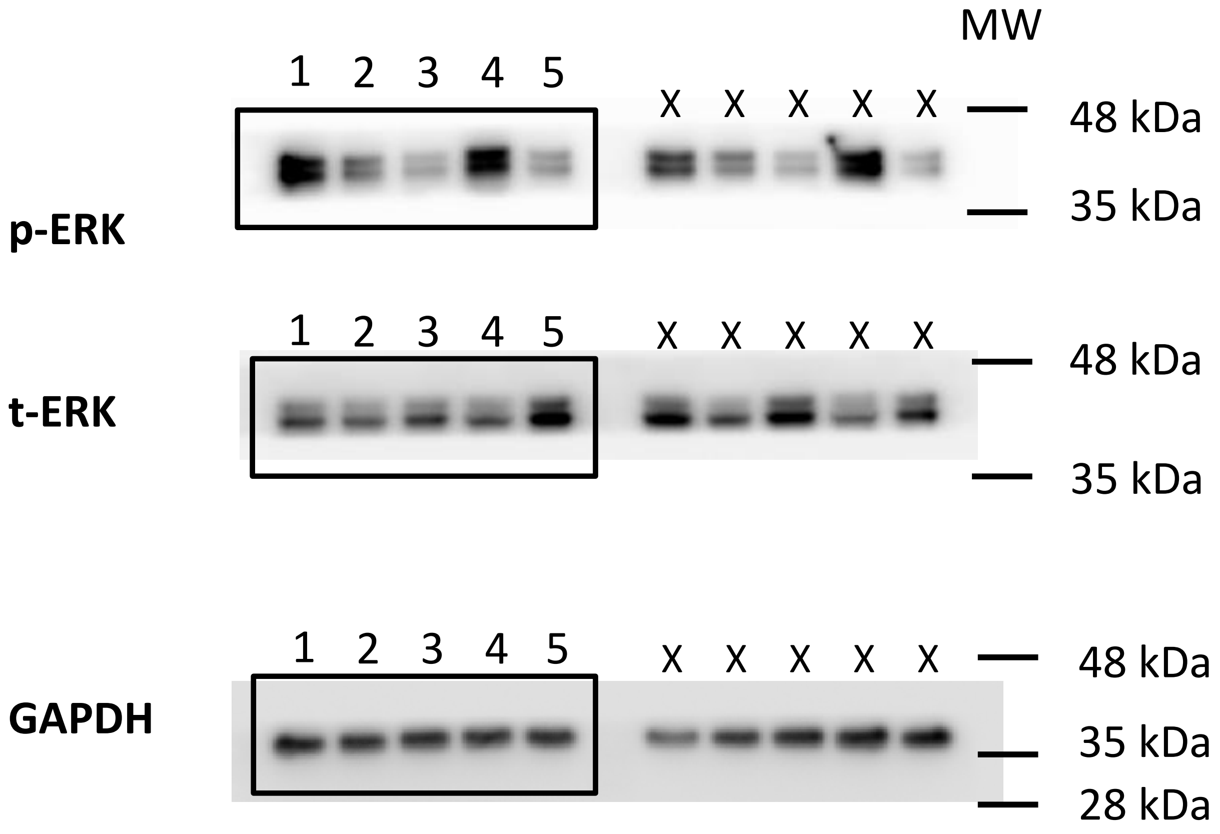

1: CTRL

2: H_2O_2

3: PD

4: H-LIPEF+H_2O_2

5: PD+H-LIPEF+H_2O_2
